# The Removal of Formaldehyde from Urea Formaldehyde Adhesive by Sodium Borohydride Treatment and Its Application in Plywood

**DOI:** 10.3390/polym16070969

**Published:** 2024-04-02

**Authors:** Xi Wang, Hui Zhao, Bo Zhang, Xiuchan Wen, Siyu Huang, Weixing Gan

**Affiliations:** 1 Key Laboratory of Ecology of Rare and Endangered Species and Environmental Protection (Guangxi Normal University), Ministry of Education, Guilin 541006, China; 2 College of Environment and Resources, Guangxi Normal University, Guilin 541004, China; 18943088627@163.com (B.Z.);

**Keywords:** urea formaldehyde adhesive, formaldehyde scavenger, sodium borohydride, plywood

## Abstract

The global production of plywood is constantly increasing as its application in the furniture and interior decoration industry becomes more widespread. An urgent issue is how to decrease the formaldehyde released from plywood, considering its carcinogenic effect on humans and harm to the environment. Reducing the free formaldehyde content of the urea formaldehyde (UF) adhesives used in the preparation process is considered an effective method. Therefore, it is necessary to identify a new type of formaldehyde scavengers. Here, the strongly reducing substance sodium borohydride was used to reduce and degrade the free formaldehyde in UF adhesives, and its effects on the properties of the UF adhesive and plywood were studied. When 0.7% sodium borohydride was added to the UF adhesive with a molar ratio of formaldehyde to urea of 1.4:1, the free formaldehyde content of the UF resin decreased to 0.21%, which is 53% lower than that of the untreated control. Moreover, the formaldehyde released from the plywood was reduced to 0.81 mg/L, ~45% lower than that from the group. The bonding strength of the treated samples could reach ~1.1 MPa, which was only reduced by ~4% compared to that of the control. This study of removing formaldehyde from UF adhesive by reduction could provide a new approach for suppressing formaldehyde release from the final products.

## 1. Introduction

Urea formaldehyde (UF) adhesives have the advantages of a high bonding strength, simple synthesis process, and low cost [1,2], and they have been widely used in plywood manufacturing. However, there are some obvious disadvantages of UF resin adhesives, such as poor water resistance, high brittleness of the cured adhesive layer [3,4], and high formaldehyde emissions, which have become long-standing challenges in the plywood industry [5]. Formaldehyde, a typical carcinogen which is released from UF resin products, is hazardous to human health for both resin producers and consumers [6,7]. In recent years, many studies have been conducted on the synthesis of UF resins, which has proposed some approaches such as optimizing the synthesis process of the adhesive, reducing the molar ratio of formaldehyde to urea (F/U), and using formaldehyde scavengers [8,9]. Although it appears to have a low cost, a lower the F/U molar ratio would obviously reduce the strength of board samples to below the permissible level, which is detrimental to the water resistance of adhesive joints [10]. Que et al. [11] demonstrated that the decrease in released formaldehyde by applying UF resins with low F/U molar ratios (0.97–1.27) led to a deterioration of the mechanical properties of the particleboard. The addition of formaldehyde scavengers to UF adhesive is a simple and effective modification method [8]. Moubarik et al. [12] achieved an ~11% reduction in formaldehyde released from plywood by adding 7% corn flour compared to a control sample. Chen et al. [13] achieved a ~28.9% reduction in formaldehyde released from plywood to 0.86 mg/L by adding ~3 wt% almond shells as a formaldehyde adsorbent to the UF resin. Cademartori et al. [14] demonstrated a substantial reduction in formaldehyde release by adding Al_2_O_3_ nanoparticles to a UF resin. Shao et al. [15] reduced the formaldehyde release from plywood by incorporating a 32 wt% attapulgite filler. However, formaldehyde is merely physically and reversibly absorbed on the aforementioned inorganic scavengers, thus the formaldehyde content in plywood is not decreased, and this physically adsorbed formaldehyde will be slowly released during storage or use of the plywood. Using organic formaldehyde scavengers in UF resin, the formaldehyde released from the particleboard was reduced by ~27.9% (by adding an ~1.0% tannin solution) but had a negative effect on bonding strength [16]. It has also been shown that the incorporation of 1% propylamine as a formaldehyde scavenger in UF resins reduced the formaldehyde released from the particleboard from ~0.7 mg/L to around ~0.3 mg/L [17]. Such organic amine formaldehyde scavengers can significantly reduce the free formaldehyde content of UF adhesives and hence suppress the release of formaldehyde released from the products, but negatively affected the bond strength.

Formaldehyde, due to its reducibility, could be oxidized by Cu^2+^ to formic acid or carbon dioxide and water in chemical copper plating. We have used Cu^2+^ as an oxidant to oxidize formaldehyde in UF adhesives into the less toxic HCOO^−^ as a method of reducing the content of free formaldehyde in UF resin adhesives. Meanwhile, formaldehyde also has weak oxidizing properties, and it can be reduced to methanol by strongly reducing substances such as sodium borohydride and lithium aluminum hydride.

In order to broaden the research field of formaldehyde scavengers, we proposed such a strongly reducing formaldehyde scavenger for use in UF adhesives.

Hence, the aim of our study was to investigate the potential of sodium borohydride as a formaldehyde scavenger in plywood. The effects of strongly reducing sodium borohydride on the properties of the UF adhesive were investigated, while using a synthetic UF adhesive with an F/U molar ratio of 1.4:1 as a control group. The modified UF adhesive samples were characterized by differential scanning calorimetry (DSC), thermogravimetry (TG)/differential thermogravimetry (DTG), and Fourier-transform infrared (FTIR) spectroscopy. This study offers a new approach for reducing the content of free formaldehyde in UF adhesives and hence suppress its release during use of the products, while maintaining good performance.

## 2. Materials and Methods

### 2.1. Materials

Ammonium chloride, urea, sodium borohydride, formaldehyde, formic acid, and sodium hydroxide (AR, Xilong Science Co., Ltd., Shantou, China) were used as raw materials. Flour was obtained from Beijing Guchuan Flour Company, Beijing, China. The eucalyptus veneer (480 mm × 480 mm × 1.7 mm; moisture content: 8–12%) used to prepare the plywood was purchased from Guilin Liding Plywood Factory, Guilin, China.

### 2.2. Equipment

Resins were prepared with a flat vulcanizer (XLB-D, Huzhou Shunli Rubber Machinery Co., Ltd., Huzhou, China). Sodium borohydride and the UF resin were mixed using the aid of a magnetic stirrer (79-1, Jiangsu Zhongda Industry Co., Ltd., Changzhou, China). The strength of the samples was measured by a universal mechanical testing machine (MWW-10A, specification 10 kN, Jinan Xinguang Testing Machine Manufacturing Co., Ltd., Jinan, China). FTIR spectra were recorded using a Spectrum One spectrometer (Perkin-Elmer, Waltham, MA, USA). TG was performed using a synchronous thermal analyzer (STA-449C, Netzsch, Selb, Germany).

### 2.3. Determination of Free Formaldehyde in UF Resin

The formaldehyde emissions from the UF resin were determined by the dryer method stipulated in “GB/T 14074-2017: Test methods for wood adhesives and their resins” [18]. The size of the specimens was 150 mm × 50 mm, and the number of specimens in each group was 10. First, distilled water (300 mL) was placed at the bottom of the dryer. Next, each group of specimens was fixed to a metal frame and placed on a glass plate containing distilled water. The dryer was then covered and placed in a humid chamber at 25 °C for 24 h. Thereafter, 25 mL of water in a glass dish was mixed with acetylacetone (25 mL) and ammonium acetate to prepare a test solution. The test solution was poured into a conical flask and placed in a water bath at 63 °C for 10 min, and then cooled to 25 °C. The absorbance of the test solution was measured at 412 nm using a UV/Vis spectrophotometer. The formaldehyde released was then determined by referencing a calibration curve prepared by analyzing standard formaldehyde solutions.

### 2.4. Experimental Methods

#### 2.4.1. Synthesis of UF Adhesive

UF adhesive (the base adhesive A) was synthesized as described in a previous report [9]. The solid content of the UF resin was 49.6%, and its viscosity equated to a drainage time of 18.6 s (vide infra).

#### 2.4.2. Modification of UF Adhesive with Sodium Borohydride

The UF adhesive (100 g) and sodium borohydride (*x* g) were mixed with the aid of a magnetic stirrer spun at 250 rpm for 20 min to obtain a sodium borohydride-modified UF adhesive (denoted as BMU-*x*, where *x* denotes the added mass of sodium borohydride).

#### 2.4.3. Viscosity Test

The viscosity of the UF adhesive was determined according to Chinese national standard GB/T 14074-2017 (“Testing methods for wood adhesives and their resins”) [18]. The requisite amount of UF adhesive was poured into a T-4 viscosity cup at 30 °C, such that the top surface of the UF adhesive was level with the top of the 4-cup apparatus. The UF adhesive was then allowed to flow out naturally from a small hole at the bottom of the T-4 viscosity cup. The outflow time of the adhesive was recorded, giving a measure of its viscosity.

#### 2.4.4. Solid Content Test

According to Chinese national standard GB/T 14074-2017 (“Testing methods for wood adhesives and their resins”) [18], the UF adhesive (1 ± 0.1 g) was placed in an aluminum foil cup and dried in an oven for 2 h at 120 °C. The solid content of the resin was calculated according to the following formula:

(1) $$C=\frac{m_{3}-m_{1}}{m_{2}-m_{1}}\times 100\%$$

where *C* is the solid content of the UF adhesive, *m*_1_ is the mass of the aluminum foil cup, *m*_2_ is the mass of the aluminum foil cup with the UF adhesive before drying, and *m*_3_ is the mass of the aluminum foil cup with the resin after drying.

#### 2.4.5. Determination of Free Formaldehyde in UF Adhesive

According to Chinese national standard GB/T 14074-2017 (“Testing methods for wood adhesives and their resins”), the content of free formaldehyde in the UF resin adhesive was determined by NaOH titration [18]. It was calculated according to the following formula:

(2) $$F\%=\frac{\left(V_{2}-V_{1}\right)\times N\times 0.03003}{G}\times 100\%$$

where *V*_1_ is the volume of the NaOH solution consumed in titrating the resin, *V*_2_ is the volume of the NaOH solution consumed in titrating the blank sample, *N* is the concentration of the NaOH standard solution, and G is the mass of the UF resin sample.

### 2.5. Plywood

#### 2.5.1. Preparation of Plywood

The composition of the UF adhesive paste used for plywood preparation was as follows: 66 g UF adhesive A, 13 g flour. and 0.66 g NH_4_Cl; these were combined in a 250 mL beaker and manually stirred by means of a glass rod to obtain the control UF adhesive paste. Using the same process, 66 g UF adhesive A was replaced by 66 g BMU-x adhesive to obtain the sodium borohydride-modified UF adhesive paste.

The plywood was prepared according to the Chinese national standard GB/T 9846-2015 (“Plywood for general use”), as previously described [19]. A total of 80 g of UF adhesive paste was coated on either side of a eucalyptus veneer (480 mm × 480 mm × 1.7 mm; water content: 8–12%) using a rubber scraper (the amount of adhesive applied on each side was 40 g, equal to 166 g/m^2^). The eucalyptus veneer coated with adhesive on both sides was placed between two eucalyptus veneers without adhesive to form a three-layer board. This three-layer board was pressed at room temperature and under 0.8 MPa for 30 min, and then at 115 °C and 1.2 MPa for 5 min [9] to obtain the final product.

#### 2.5.2. Performance Tests of Plywood

The size of the plywood specimens was 100 mm × 25 mm, the length of the shear surface was 25 mm, and the depth of the cut was two-thirds of the total thickness. The specimens were immersed in water at 63 °C for 3 h and then air-dried for 20 min. According to the requirements of “GB/T 17657-2013: Test methods for evaluating the properties of wood-based panels and surface-decorated wood-based panels” [20], the bonding strengths of wood-based panels and surface-decorated panels were tested on a microcomputer-controlled electronic universal testing machine. The two ends of the specimen were clamped on the pair of jigs of the universal testing machine, and the distance between the clamped parts and the notch was less than 5 mm. To ensure the validity of the test results, at least six samples of each plywood specimen were tested and then the averaged values were calculated.

### 2.6. Performance Tests of UF Resins

The base adhesive A and BMU-0.7 were freeze-dried at −70 °C for 24 h, and then ground into powders to obtain the UF resins (samples B and sample C).

#### 2.6.1. FTIR Characterization of UF Resins

Potassium bromide and the UF resins (samples B and sample C) were homogeneously mixed, and then the mixtures were pressed into sheets. The FTIR spectra of these tablets was recorded on a Perkin-Elmer Spectrum One spectrometer over the wavenumber range of 400–4000 cm^−1^ with a resolution of 4 cm^−1^.

#### 2.6.2. TG Analysis of UF Resins

TG tests of the UF resins (samples B and sample C) were carried out using a STA-449C thermal analyzer over the range of 30–600 °C at a heating rate of 10 °C min^−1^ under a N_2_ atmosphere.

#### 2.6.3. DSC Analysis of UF Resins

DSC tests on the UF resins (samples B and sample C) were carried out using the STA-449C thermal analyzer over the range of 30–200 °C at a heating rate of 10 °C·min^−1^. The protective gas was N_2_ at a flow rate of 60 mL·min^−1^.

### 2.7. Statistical Analysis

The bonding strength of the plywood was investigated by using one-way analysis of variance (ANOVA) with a 95% confidence level. The statistical analyses were performed using the statistical software SPSS (Version 27.0; IBM, Armonk, NY, USA).

## 3. Results

### 3.1. Effects of Sodium Borohydride Addition on the Properties of UF Resin Adhesive

To study the effects of the addition of sodium borohydride on the bonding strength and the release of formaldehyde from plywood prepared with UF resin, plywood samples were prepared with the base adhesive A and the modified UF adhesives BMU-0.5, BMU-0.7, BMU-1, and BMU-1.5, and then their properties were compared.

#### 3.1.1. Effect of Addition of Sodium Borohydride on Free Formaldehyde Content of UF Adhesive

**Figure 1:**
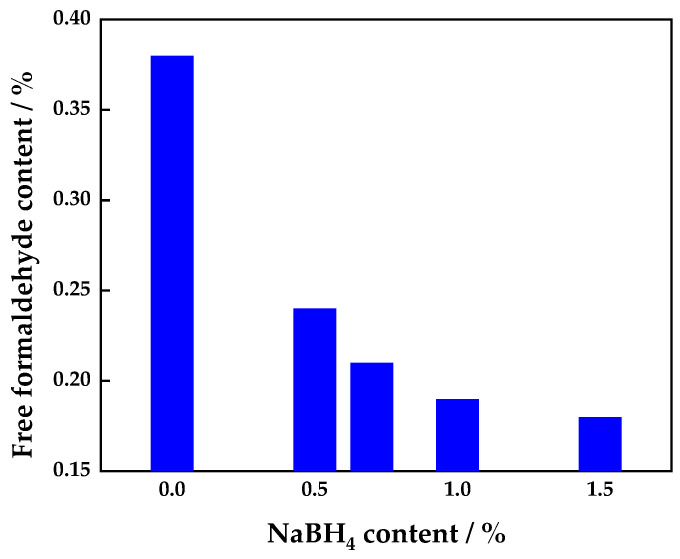
Relationship between the sodium borohydride content in UF resin adhesive and the free formaldehyde content.

Figure 1 shows the effect of sodium borohydride loading on the free formaldehyde content of the UF adhesive. The free formaldehyde content of the base adhesive A without sodium borohydride (the control UF adhesive) was about 0.45%, as we have reported previously. The free formaldehyde content in the UF adhesive modified with 0.5% sodium borohydride was 0.24%, that is, around 47% lower than that of the control UF adhesive, and considerably lower than the value of 0.3% stipulated by Chinese standard GB/T 14732-2017 for UF adhesives used in plywood [21]. This could be ascribed to the chemical reduction of free formaldehyde by sodium borohydride to form the less toxic methanol. A strongly reducing H^–^ ion from sodium borohydride can be envisaged as reacting with free formaldehyde in the UF adhesive, followed by hydrolysis (acidolysis) to generate methanol and boric acid. The possible chemical reaction equations are as follows [22]:

NaBH_4_ + 4R_2_CO → NaB(OCHR_2_)_4_ (3)

B(OCHR_2_)_4_^−^ + 2OH^−^ + H_2_O → 4R_2_CHOH + BO_3_^3−^ (4)

The free formaldehyde content in the modified UF adhesive decreased with increasing sodium borohydride content. When 0.7% sodium borohydride was added to the UF resin adhesive, the free formaldehyde content was 0.21%. Compared to the control UF resin, the free formaldehyde content was reduced by 53%, which exceeded the removal rate of free formaldehyde from the UF resin when using amine as the scavenger (50%) [23].

#### 3.1.2. Effect of Sodium Borohydride Content on the Bonding Strength of Plywood

**Figure 2:**
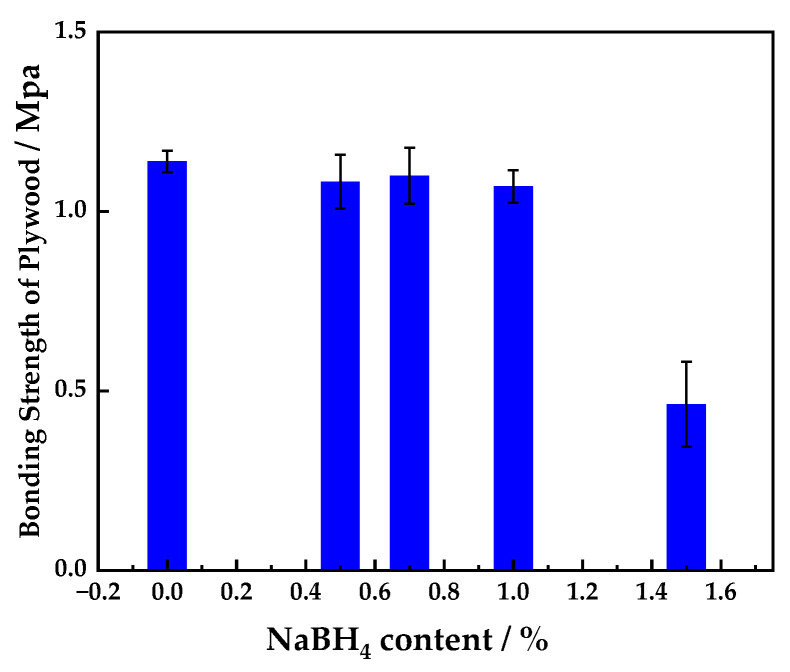
Relationship between the sodium borohydride content in UF resin adhesive and the bonding strength of the prepared plywood.

Figure 2 shows the relationship between the sodium borohydride content in the UF resin adhesive and the bonding strength of the prepared plywood. It can be seen from Figure 2 that the bonding strength of the plywood prepared with the control UF adhesive (the base adhesive A) was 1.14 MPa. The bonding strength of the plywood prepared with the UF adhesive modified with 0.5% sodium borohydride was 1.08 MPa, that is, around 5% lower than that of the plywood prepared with the control UF adhesive. The bonding strength of the plywood prepared with UF adhesive modified with 0.7% sodium borohydride was 1.1 MPa, around 4% lower than that of the control. The decrease in strength can be attributed to the formation of methanol when sodium borohydride is added to the UF resin [22]. The methanol will react with sodium borohydride to produce hydrogen, which will reduce the cross-linking density of the resin, thus reducing the bonding strength of the prepared plywood. Indeed, the bonding strength of the prepared plywood decreased with the increase in sodium borohydride content in the range of 0–1.5%. The greater the loading of sodium borohydride used to modify the UF resin, the more hydrogen will be produced by its reaction with methanol, leading to a greater reduction in the cross-linking degree of the UF resin, causing the bonding strength of the UF resin products to decline more severely. The bonding strength of the plywood prepared with the UF adhesive modified with 1.5% sodium borohydride was 0.46 MPa, around 60% lower than that of plywood prepared with the control UF adhesive. ANOVA tests (95% confidence) showed that the addition of different percentages of sodium borohydride to the wood adhesives had a significant effect on plywood strength.

#### 3.1.3. Effect of Sodium Borohydride Content on Release of Formaldehyde from Plywood

**Figure 3:**
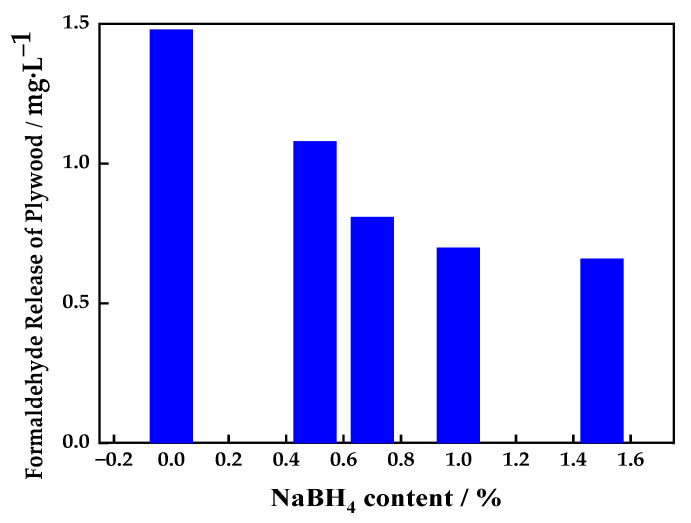
Relationship between sodium borohydride content in UF resin adhesive and formaldehyde released from plywood.

Figure 3 shows the relationship between sodium borohydride content in the UF adhesive and the formaldehyde released from the plywood. It can be seen from Figure 3 that the formaldehyde released from the plywood prepared with the control UF adhesive was 1.48 mg/L, within the range of level E1 (≤1.5 mg/L) according to the Chinese release standard (GB/T 18580-2001) [24], although at the high end of this range. With the addition of 0.5% and 0.7%sodium borohydride, the formaldehyde released from the plywood samples was 1.08 mg/L and 0.81 mg/L, respectively, which are 27% and 45% lower than that from the plywood prepared with the control UF adhesive. Thus, the amount of formaldehyde released decreased with increasing sodium borohydride. When 1.5% sodium borohydride was added, the formaldehyde released from the plywood was 0.66 mg/L, which is 55% lower than that from the control.

**Figure 4:**
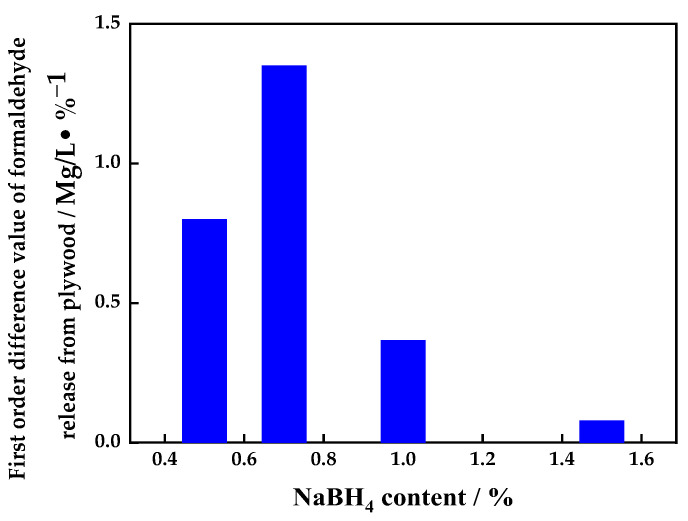
Relationship between sodium borohydride content in UF adhesive and first-order difference value of formaldehyde release from plywood.

Figure 4 shows the relationship between the sodium borohydride content in the UF adhesive and the first-order difference value of the formaldehyde released from the plywood. It can be seen from Figure 4 that the first-order difference value of the formaldehyde released from the plywood had a maximum value of 1.35 mg·L^−1^·(% NaBH_4_)^−1^ for plywood prepared using the UF adhesive modified with 0.7% sodium borohydride, reflecting the best inhibitory effect.

Taking into account the cost of the adhesive, the mechanical properties of the plywood, the amount of formaldehyde release, and the first-order difference in the formaldehyde released, 0.7% sodium borohydride was selected as the optimal loading as a UF adhesive modifier.

### 3.2. FTIR Characterization of UF Resin

**Figure 5:**
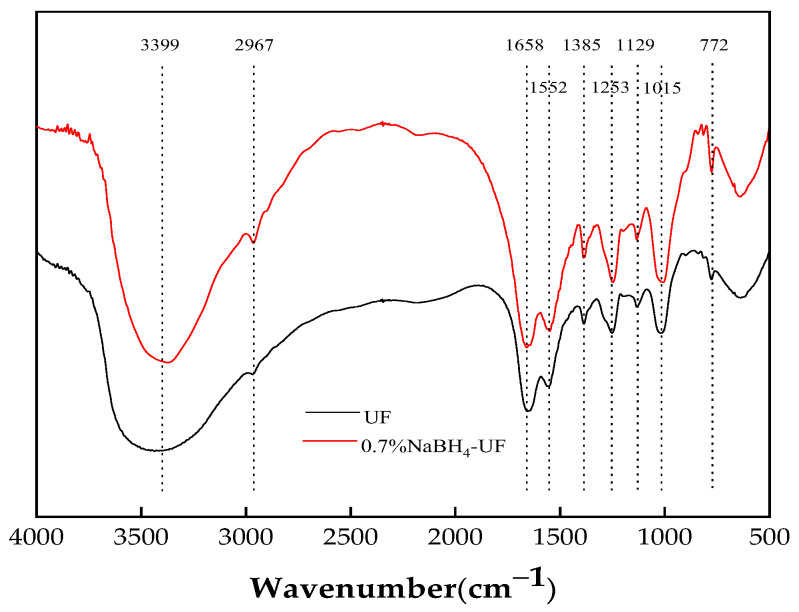
FTIR spectra of pure UF resin and 0.7% NaBH_4_-UF resin.

Figure 5 shows the FTIR spectra of the UF resin before and after adding sodium borohydride (0.7% NaBH_4_-UF adhesive). It can be seen from Figure 5 that the FTIR spectra of the control UF adhesive (pure UF resin) and the UF resin incorporating 0.7% sodium borohydride (0.7% NaBH_4_-UF adhesive) were basically the same. The broad peak at 3300–3500 cm^−1^ can be attributed to the stretching vibrations of O–H and N–H bonds [25]. The peak at 2800–3000 cm^−1^ can be attributed to asymmetric C–H stretching vibrations [26]. The peak at 1655–1680 cm^−1^ can be attributed to the C=O stretching vibration of the aliphatic aldehyde group [27]. The peak at 1530–1560 cm^−1^ can be attributed to a –C–NH bending vibration [28]. The peak at 1380 cm^−1^ can be attributed to the stretching vibration of the C–N bond. The peak at 1250 cm^−1^ can be attributed to a stretching vibration of the C–O bond in –COOH [29]. Further peaks at 1150–1060 cm^−1^, 1000–1015 cm^−1^, and 770–785 cm^−1^ may be due to C–O–C and C–O–H stretching vibrations and an in-plane rocking vibration of –CH_2−_, respectively [9,30,31,32].

In Figure 5, it is evident that the characteristic peaks of sodium borohydride at 2387, 2292, and 2217 cm^−1^ were absent from the spectrum of the 0.7% NaBH_4_-UF resin, indicating that the sodium borohydride added has completely reacted with species such as formaldehyde in the UF adhesive.

It can be seen that the peak at 3399 cm^−1^ of the 0.7% NaBH_4_-UF resin was more intense than that of the control UF adhesive. This may be due to the stretching vibration of an intermolecular hydrogen bond (O–H···O) of boric acid formed by the oxidation of sodium borohydride by formaldehyde [22]. These results are consistent with the free formaldehyde content being reduced by adding sodium borohydride to the UF adhesive.

### 3.3. Thermogravimetric Analysis of UF Resin

The thermal stability of a resin can better reflect its basic properties, such as weather resistance and aging resistance. A thermogravimetric analysis (TGA) can be used to determine the molecular weight and thermal stability of a polymer. As the temperature is gradually increased, some small molecules in the resin will evaporate, and the least stable chemical bonds in the resin will break down. Subsequently, the stable chemical bonds will also break down, leading to thermal degradation and carbonization.

**Figure 6:**
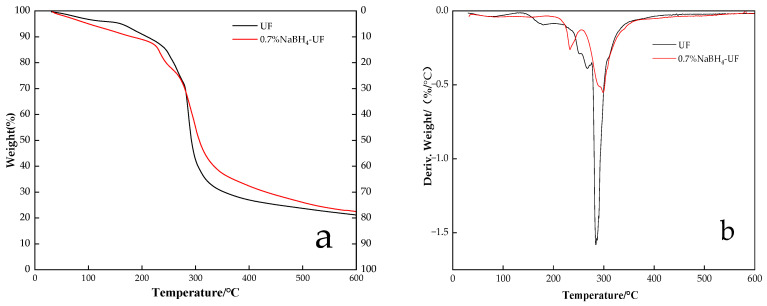
TG (**a**) and DTG (**b**) traces of pure UF resin and 0.7% NaBH_4_-UF resin.

Figure 6 shows the TG (a) and DTG (b) traces of the UF resins (pure UF and 0.7% NaBH_4_-UF resins). The thermal degradation process of the UF adhesive can be divided into three main stages:

1. The first stage, in the range of 30–200 °C, was the evaporation of residual water and the release of formaldehyde from the UF resin, as well as the removal of water and formaldehyde produced by the condensation reaction of methylol and amine groups [33]. It can be seen from Figure 6a that the mass loss in the range of 30–200 °C from the control UF adhesive (9.2%) was lower than that from the 0.7% NaBH_4_-UF adhesive (11.1%). This may indicate that there was more water lost from hydrated boric acid during thermal decomposition at 30–200 °C (boric acid produced by reaction of sodium borohydride with formaldehyde [22]) in the 0.7% NaBH_4_-UF adhesive.
2. The second stage, in the range of 200–350 °C, involved mass loss due to the breaking and decomposition of dimethyl ether and dimethyl bonds in the resin [34]. From the DTG traces, it can be seen that decomposition of the resin was extensive in this stage. The temperatures of maximum pyrolysis of the pure UF resin and 0.7% NaBH_4_-UF resin were determined to be 294 °C and 299 °C, respectively. Above 280 °C, the mass loss from the 0.7% NaBH_4_-UF resin was less than that from the pure UF resin.
3. The third mass loss stage, in the range of 350–600 °C, involved the oxidative elimination of nitrogen, oxygen, hydrogen, and other elements, such that the resin was carbonized [29]. The amount of pyrolysis residue from the pure UF (21%) was lower than that from the 0.7% NaBH_4_-UF (23%) after the third stage at 600 °C. This may be due to the boron oxide formed by thermal decomposition of the boric acid that was produced by the reaction of sodium borohydride with formaldehyde. Boron oxide compounds can increase the amount of thermal decomposition residue from a resin in two ways: (1) they are inorganic compounds with low thermal decomposition and volatilization losses below 600 °C so the presence of these boron oxide compounds increases the amount of residue; (2) the presence of inorganic oxide particles creates a shielding effect. Their presence does not necessarily represent an increase in thermal stability.

**Figure 7:**
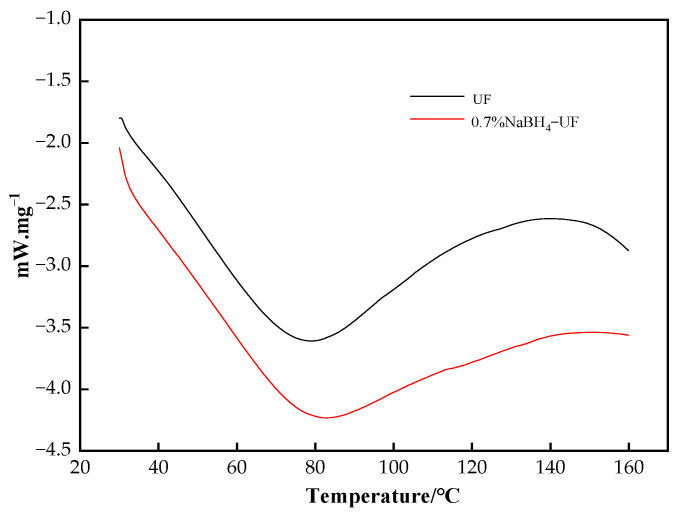
DSC traces of UF resin and 0.7% NaBH_4_-UF resin (heating rate 10 °C·min^−1^).

Figure 7 shows the DSC traces of the UF resin and 0.7% NaBH_4_-UF resin. The DSC traces of both the 0.7% NaBH_4_ UF resin and pure UF resin exhibited an exothermic peak. The curing peak temperature of the 0.7% NaBH_4_-UF resin was 1.8 °C higher than that of the pure UF resin, and the corresponding enthalpy value was 32.20 J·g^−1^ higher. Hence, with the addition of sodium borohydride, resin curing became more difficult and the heat release increased.

## 4. Conclusions

The effects of sodium borohydride on the properties of a UF resin were studied in detail. The free formaldehyde content and release of formaldehyde from the plywood decreased with increasing sodium borohydride content in the range of 0–1.5 wt%. At the same time, the addition of sodium borohydride had a detrimental effect on the bonding strength of the plywood.

The first-order difference value of the formaldehyde released from the plywood showed a maximum at a sodium borohydride loading of 0.7%. The free formaldehyde content in this sample was 0.21%, and the formaldehyde released from the plywood was 0.81 mg/L. Compared to the control UF resin, the free formaldehyde content was reduced by 53% and the formaldehyde released from the plywood was reduced by 45%. The bonding strength of this plywood sample was 1.1 MPa, around 4% lower than that of the plywood prepared with the control UF adhesive.

The FTIR analysis corroborated that the weak oxidant formaldehyde in the adhesive could be reduced and removed by the strong reducing agent sodium borohydride. The TGA also demonstrated that the curing peak temperature and enthalpy of the UF resin were increased and its heat resistance was improved by loading with sodium borohydride. That is to say, it became more difficult to be cured and its thermal stability was improved.

## Data Availability

Data are contained within the article.
